# Supplementary material for: Farming practices affect the amino acid profiles of the aquaculture Chinese mitten crab
Source: PeerJ. 2021 Jun 25;9:e11605. doi: 10.7717/peerj.11605 (PMC8237828; doi:10.7717/peerj.11605)
Supplement: Supplemental Information 2 — Different letters represent significant differences among sites for p < 0.05. Asp, Aspartic acid; Thr, Threonine; Ser, Serine; Glu, Glutamic acid; Gly, Glycine; Ala, Alanine; Cys, Cystine; Val, Valine; Met, Methionine; Ile, Isoleucine; Leu, Leucine; Tyr, Tyrosine; Phe, Phenylalanine; Lys, Lysine; His, Histidine; Arg, Arginine; Pro, Proline. TAA, total amino acids; EAA, essential amino acids; NEAA, non-essential amino acids; DAA, delicious amino acids; EAA/TAA, essential amino acids/total amino acids. [file peerj-09-11605-s002.docx]

Table S1 Amino acid concentration (g 100g^-1^ of dry weight) of mitten crab hepatopancreas from each site and sex (mean± SE).

|  | Suqian | | Guannan | | Sihong | | Xinghua | | Gaochun | | Jintan | | Suzhou | | |
| --- | --- | --- | --- | --- | --- | --- | --- | --- | --- | --- | --- | --- | --- | --- | --- |
|  | ♂ | ♀ | ♂ | ♀ | ♂ | ♀ | ♂ | ♀ | ♂ | ♀ | ♂ | ♀ | ♂ | ♀ | |
| Asp | 1.11±0.18b | 0.94±0.03b | 1.33±0.01a | 1.25±0.03a | 1.01±0.04b | 1.17±0.04b | 0.86±0.02b | 1.07±0.03b | 1.24±0.02b | 0.94±0.01b | 1.15±0.04b | 0.94±0.02b | 1.41±0.04ab | 1.06±0.05ab |  |
| Thr | 0.58±0.04b | 0.48±0.04b | 0.76±0.05a | 0.68±0.03a | 0.59±0.03ab | 0.67±0.01ab | 0.47±0.03b | 0.55±0.02b | 0.72±0.04ab | 0.55±0.03ab | 0.65±0.01b | 0.52±0.04b | 0.74±0.01ab | 0.59±0.05ab |  |
| Ser | 0.45±0.01c | 0.37±0.04c | 0.54±0.04a | 0.5±0.05a | 0.46±0.02b | 0.5±0.07b | 0.35±0.02c | 0.43±0.03c | 0.56±0.02b | 0.42±0.02b | 0.5±0.04bc | 0.4±0.05bc | 0.59±0.09ab | 0.47±0.03ab |  |
| Glu | 1.24±0.02c | 1.08±0.08c | 1.62±0.02a | 1.52±0.02a | 1.26±0.01b | 1.41±0.05b | 1.03±0.03c | 1.2±0.06c | 1.57±0.03b | 1.16±0.04b | 1.38±0.01bc | 1.11±0.01bc | 1.63±0.07ab | 1.29±0.04ab |  |
| Gly | 0.65±0.06b | 0.49±0.07b | 0.78±0.04a | 0.68±0.04a | 0.62±0.05a | 0.69±0.04a | 0.44±0.01b | 0.56±0.01b | 0.66±0.04ab | 0.53±0.03ab | 0.66±0.02b | 0.49±0.04b | 0.77±0.04a | 0.57±0.03a |  |
| Ala | 0.83±0.03bc | 0.55±0.05bc | 1.09±0.06a | 0.89±0.07a | 0.87±0.02ab | 0.87±0.04ab | 0.52±0.02c | 0.77±0.03c | 0.9±0.05b | 0.71±0.04b | 0.82±0.02bc | 0.63±0.03bc | 1.04±0.04ab | 0.73±0.03ab |  |
| Cys | 0.21±0.03b | 0.17±0.01b | 0.23±0.03a | 0.24±0.04a | 0.23±0.03a | 0.29±0.03a | 0.17±0.04b | 0.18±0.04b | 0.34±0.01a | 0.2±0.02a | 0.23±0.04a | 0.18±0.03a | 0.37±0.03a | 0.23±0.02a |  |
| Val | 0.56±0.01b | 0.47±0.03b | 0.66±0.03a | 0.65±0.01a | 0.55±0.01ab | 0.66±0.02ab | 0.45±0.05b | 0.53±0.03b | 0.67±0.03ab | 0.52±0.02ab | 0.59±0.04b | 0.49±0.06b | 0.73±0.03ab | 0.55±0.03ab |  |
| Met | 0.26±0.02 | 0.22±0.04 | 0.3±0.01 | 0.29±0.04 | 0.25±0.03 | 0.31±0.04 | 0.2±0.03 | 0.25±0.03 | 0.27±0.03 | 0.23±0.03 | 0.26±0.03 | 0.21±0.01 | 0.33±0.03 | 0.24±0.03 |  |
| Ile | 0.49±0.02ab | 0.39±0.01ab | 0.56±0.05a | 0.52±0.04a | 0.45±0.04ab | 0.49±0.04ab | 0.35±0.05b | 0.44±0.03b | 0.54±0.04ab | 0.42±0.04ab | 0.48±0.02ab | 0.39±0.02ab | 0.6±0.02a | 0.45±0.02a |  |
| Leu | 0.83±0.03b | 0.67±0.01b | 0.97±0.03a | 0.9±0.03a | 0.79±0.11ab | 0.87±0.03ab | 0.61±0.04b | 0.76±0.06b | 0.96±0.01ab | 0.74±0.01ab | 0.83±0.03b | 0.66±0.01b | 1.01±0.01a | 0.81±0.01a |  |
| Tyr | 0.44±0.02ab | 0.35±0.03ab | 0.46±0.04a | 0.41±0.01a | 0.39±0.04ab | 0.4±0.03ab | 0.18±0.02b | 0.31±0.01b | 0.42±0.02b | 0.25±0.03b | 0.36±0.03b | 0.22±0.02b | 0.52±0.03ab | 0.28±0.05ab |  |
| Phe | 0.53±0.05b | 0.48±0.05b | 0.64±0.02ab | 0.62±0.02ab | 0.51±0.05ab | 0.62±0.02ab | 0.42±0.02b | 0.51±0.02b | 0.67±0.03ab | 0.49±0.08ab | 0.57±0.01b | 0.47±0.06b | 0.78±0.01a | 0.54±0.04a |  |
| Lys | 0.75±0.01bc | 0.6±0.05bc | 0.88±0.03a | 0.82±0.04a | 0.69±0.07ab | 0.8±0.04ab | 0.47±0.03c | 0.63±0.02c | 0.81±0.01b | 0.62±0.04b | 0.72±0.02bc | 0.56±0.06bc | 0.92±0.02ab | 0.68±0.03ab |  |
| His | 0.28±0.07 | 0.23±0.03 | 0.34±0.04 | 0.34±0.04 | 0.24±0.01 | 0.3±0.05 | 0.18±0.02 | 0.26±0.02 | 0.3±0.05 | 0.26±0.03 | 0.26±0.03 | 0.22±0.02 | 0.35±0.03 | 0.27±0.02 |  |
| Arg | 0.78±0.04b | 0.67±0.01b | 0.96±0.02a | 0.83±0.03a | 0.83±0.03a | 0.94±0.04a | 0.54±0.04b | 0.75±0.03b | 0.82±0.02b | 0.64±0.03b | 0.85±0.02b | 0.62±0.02b | 1.03±0.03a | 0.72±0.02a |  |
| Pro | 0.59±0.01b | 0.41±0.01b | 0.73±0.03a | 0.59±0.04a | 0.78±0.05a | 0.71±0.04a | 0.43±0.03b | 0.45±0.04b | 0.51±0.04b | 0.43±0.03b | 0.56±0.02b | 0.39±0.04b | 0.84±0.04a | 0.54±0.03a |  |
| TAA | 10.57±0.65b | 8.57±0.6b | 12.84±0.55a | 11.74±0.58a | 10.5±0.42ab | 11.7±0.63ab | 7.64±0.5b | 9.64±0.51b | 11.96±0.49ab | 9.1±0.53ab | 10.85±0.43b | 8.48±0.54b | 13.67±0.57a | 10.01±0.53a | |
| EAA | 4.01±0.18b | 3.31±0.23b | 4.77±0.22a | 4.48±0.21a | 3.82±0.12ab | 4.42±0.2ab | 2.96±0.25b | 3.67±0.21b | 4.65±0.19ab | 3.56±0.25ab | 4.1±0.16b | 3.29±0.26b | 5.11±0.13a | 3.86±0.21a |  |
| NEAA | 6.57±0.47b | 5.26±0.37b | 8.07±0.33a | 7.26±0.37a | 6.68±0.3ab | 7.28±0.43ab | 4.69±0.25b | 5.97±0.3b | 7.31±0.3b | 5.54±0.28b | 6.76±0.27b | 5.19±0.28b | 8.55±0.44a | 6.15±0.32a |  |
| DAA | 3.82±0.29bc | 3.06±0.23bc | 4.82±0.13a | 4.34±0.16a | 3.75±0.12b | 4.15±0.17b | 2.85±0.08c | 3.6±0.13c | 4.37±0.14b | 3.35±0.12b | 4±0.09bc | 3.17±0.1bc | 4.85±0.19ab | 3.64±0.15ab |  |
| EAA/TAA | 0.38 | 0.39 | 0.37 | 0.38 | 0.36 | 0.38 | 0.39 | 0.38 | 0.39 | 0.39 | 0.38 | 0.39 | 0.37 | 0.39 |  |

Different letters represent significant differences among sites for *p*<0.05.

Asp, Aspartic acid; Thr, Threonine; Ser, Serine; Glu, Glutamic acid; Gly, Glycine; Ala, Alanine; Cys, Cystine; Val, Valine; Met, Methionine; Ile, Isoleucine; Leu, Leucine; Tyr, Tyrosine; Phe, Phenylalanine; Lys, Lysine; His, Histidine; Arg, Arginine; Pro, Proline. TAA, total amino acids; EAA, essential amino acids; NEAA, non-essential amino acids; DAA, delicious amino acids; EAA/TAA, essential amino acids/total amino acids.

Table S2 Amino acid concentration (g 100g^-1^ of dry weight) of mitten crab gonad from each site and sex (mean± SE).

|  | Suqian | | Guannan | | Sihong | | Xinghua | | Gaochun | | Jintan | | Suzhou | |  |
| --- | --- | --- | --- | --- | --- | --- | --- | --- | --- | --- | --- | --- | --- | --- | --- |
|  | ♂ | ♀ | ♂ | ♀ | ♂ | ♀ | ♂ | ♀ | ♂ | ♀ | ♂ | ♀ | ♂ | ♀ | |
| Asp | 7.4±0.08a | 5.06±0.15a | 6.92±0.05b | 4.93±0.1b | 6.94±0.09b | 4.77±0.06b | 6.94±0.07b | 4.77±0.04b | 6.89±0.09b | 4.84±0.09b | 7.17±0.06b | 4.55±0.03b | 7.02±0.13a | 4.89±0.01a | |
| Thr | 5.06±0.2 | 2.92±0.06 | 4.85±0.1 | 3.15±0.06 | 5.45±0.05 | 3.06±0.88 | 5.45±0.04 | 3.06±0.91 | 5.25±0.04 | 3.11±0.04 | 5.45±0.03 | 2.9±0.04 | 5.29±0.07 | 3.07±0.05 | |
| Ser | 2.86±0.06 | 3.12±0.02 | 2.77±0.12 | 3.28±0.08 | 3.03±0.03 | 3.12±0.06 | 3.03±0.92 | 3.12±0.05 | 2.88±0.04 | 3.11±0.09 | 3.02±0.04 | 2.94±0.08 | 2.91±0.02 | 3.12±0.02 | |
| Glu | 7.69±0.11b | 6.53±0.05b | 7.88±0.08a | 7.04±0.1a | 7.88±0.1ab | 6.82±0.05aba | 7.88±0.06ab | 6.82±0.03ab | 7.98±0.08a | 6.99±0.04a | 7.32±0.07b | 6.51±0.06b | 7.86±0.01a | 7.03±0.08a | |
| Gly | 2.37±0.07a | 2.43±0.03a | 2.28±0.09a | 2.31±0.1a | 2.19±0.07a | 2.21±0.01a | 2.19±0.04a | 2.21±0.03a | 2.14±0.01a | 2.35±0.02a | 2.27±0.07b | 2.08±0.12b | 2.15±0.04a | 2.26±0.02a | |
| Ala | 4.35±0.05b | 3.24±0.04b | 4.68±0.03a | 3.68±0.01a | 4.8±0.05a | 3.55±0.05a | 4.8±0.09a | 3.55±0.05a | 4.56±0.04a | 3.66±0.08a | 4.95±0.01ab | 2.92±0.04ab | 4.59±0.04a | 3.55±0.04a | |
| Cys | 1.42±0.12 | 0.87±0.12 | 1.49±0.04 | 1.07±0.07 | 1.69±0.06 | 0.93±0.07 | 1.69±0.03 | 0.93±0.06 | 1.54±0.06 | 1±0.07 | 1.8±0.04 | 0.88±0.04 | 1.67±0.04 | 0.97±0.04 | |
| Val | 1.98±0.11 | 2.99±0.14 | 2.13±0.08 | 3.3±0.03 | 1.99±0.05 | 3.2±0.09 | 1.99±0.03 | 3.2±0.07 | 2.11±0.03 | 3.29±0.07 | 2.06±0.06 | 3.07±0.09 | 1.95±0.06 | 3.28±0.06 | |
| Met | 0.66±0.06a | 1.67±0.07a | 0.65±0.02a | 1.65±0.11a | 0.46±0.04a | 1.58±0.04a | 0.46±0.04a | 1.58±0.03a | 0.41±0.03b | 1.55±0.02b | 0.53±0.08a | 1.49±0.05a | 0.44±0.04a | 1.58±0.03a | |
| Ile | 2.69±0.12 | 2.47±0.08 | 2.74±0.04 | 2.69±0.09 | 2.8±0.09 | 2.59±0.05 | 2.8±0.07 | 2.59±0.04 | 2.66±0.04 | 2.62±0.02 | 2.9±0.04 | 2.44±0.04 | 2.68±0.02 | 2.64±0.02 | |
| Leu | 3.9±0.04 | 4.01±0.11 | 3.96±0.13 | 4.36±0.06 | 4.07±0.07 | 4.22±0.02 | 4.07±0.21 | 4.22±0.02 | 3.89±0.04 | 4.29±0.04 | 4.25±0.03 | 4.04±0.1 | 3.97±0.03 | 4.34±0.04 | |
| Tyr | 1.86±0.06a | 2.27±0.06a | 2.08±0.13a | 2.41±0.04a | 1.79±0.08b | 2.24±0.03b | 1.79±0.02b | 2.24±0.04b | 1.75±0.03a | 2.33±0.03a | 2.05±0.08b | 1.99±0.05b | 1.8±0.03b | 2.18±0.07b | |
| Phe | 2.24±0.03 | 2.63±0.06 | 2.21±0.01 | 2.65±0.11 | 2.08±0.19 | 2.51±0.04 | 2.08±0.05 | 2.51±0.05 | 1.95±0.02 | 2.52±0.03 | 2.26±0.03 | 2.41±0.05 | 2.01±0.06 | 2.47±0.02 | |
| Lys | 2.98±0.12a | 3.52±0.06a | 2.88±0.04a | 3.44±0.04a | 2.6±0.03b | 3.25±0.06b | 2.6±0.05b | 3.25±0.04b | 2.8±0.06aba | 3.35±0.03ab | 2.77±0.07b | 3.16±0.02b | 2.65±0.03ab | 3.48±0.02ab | |
| His | 1.33±0.03 | 1.07±0.12 | 1.36±0.02 | 1.08±0.21 | 1.32±0.04 | 1.02±0.09 | 1.32±0.05 | 1.02±0.92 | 1.5±0.08 | 1.14±0.01 | 1.62±0.04 | 1.05±0.05 | 1.47±0.04 | 1.11±0.01 | |
| Arg | 2.64±0.07a | 3.89±0.15a | 2.72±0.06a | 3.83±0.07a | 2.35±0.05ab | 3.76±0.08ab | 2.35±0.05ab | 3.76±0.05ab | 2.32±0.04b | 3.64±0.02b | 2.42±0.07b | 3.52±0.05b | 2.28±0.05b | 3.75±0.05b | |
| Pro | 7.95±0.09b | 2.22±0.02b | 6.44±0.03bc | 2.31±0.05bc | 7.68±0.02b | 2.36±0.08b | 7.68±0.06b | 2.36±0.03b | 7.37±0.04b | 2.47±0.03b | 8.21±0.05a | 2.42±0.03a | 7.6±0.05b | 2.55±0.05b | |
| TAA | 59.37±1.42 | 50.9±1.34 | 58.04±1.07 | 53.18±1.33 | 59.1±1.11 | 51.2 | 59.1±0.04 | 51.2±1.2 | 57.98±0.77 | 52.26±0.73 | 61.05±0.87 | 48.37±0.94 | 58.33±0.76 | 52.25±0.63 | |
| EAA | 19.5±0.68 | 20.21±0.58 | 19.42±0.42 | 21.24±0.5 | 19.44±0.52 | 20.41±0.58 | 19.44±0.49 | 20.41±0.66 | 19.06±0.26 | 20.73±0.25 | 20.22±0.34 | 19.5±0.39 | 18.99±0.31 | 20.85±0.24 | |
| NEAA | 39.87±0.74 | 30.69±0.76 | 38.62±0.65 | 31.94±0.83 | 39.66±0.59 | 30.78±0.58 | 39.66±0.45 | 30.78±0.54 | 38.92±0.51 | 31.53±0.48 | 40.83±0.53 | 28.87±0.55 | 39.34±0.45 | 31.41±0.39 | |
| DAA | 21.81±0.31a | 17.25±0.27a | 21.77±0.25a | 17.97±0.31a | 21.8±0.31a | 17.36±0.17a | 21.8±0.26a | 17.36±0.15a | 21.57±0.22a | 17.84±0.23a | 21.71±0.21b | 16.07±0.25b | 21.62±0.22a | 17.73±0.15a | |
| EAA/TAA | 0.33 | 0.4 | 0.33 | 0.4 | 0.33 | 0.4 | 0.33 | 0.4 | 0.33 | 0.4 | 0.33 | 0.4 | 0.33 | 0.4 | |

Different letters represent significant differences among sites for *p*<0.05.

Asp, Aspartic acid; Thr, Threonine; Ser, Serine; Glu, Glutamic acid; Gly, Glycine; Ala, Alanine; Cys, Cystine; Val, Valine; Met, Methionine; Ile, Isoleucine; Leu, Leucine; Tyr, Tyrosine; Phe, Phenylalanine; Lys, Lysine; His, Histidine; Arg, Arginine; Pro, Proline. TAA, total amino acids; EAA, essential amino acids; NEAA, non-essential amino acids; DAA, delicious amino acids; EAA/TAA, essential amino acids/total amino acids.

Table S3 Main composition in trash fish and commercial pellet feed of mitten crab (%)

|  | Crude protein | Crude fat | Ash |
| --- | --- | --- | --- |
| Trash fish | 64.2±0.35 | 13.6±0.26 | 15.6±0.26 |
| Commercial pellet feed | 42.6±0.55 | 9.83±0.20 | 11.33±0.18 |
